# Small-scale, homelike facilities versus regular psychogeriatric nursing home wards: a cross-sectional study into residents' characteristics

**DOI:** 10.1186/1472-6963-10-30

**Published:** 2010-01-29

**Authors:** Hilde Verbeek, Sandra MG Zwakhalen, Erik van Rossum, Ton Ambergen, Gertrudis IJM Kempen, Jan PH Hamers

**Affiliations:** 1 School for Public Health and Primary Care: CAPHRI; Faculty of Health, Medicine and Life Sciences; Department of Health Care and Nursing Science; Maastricht University; the Netherlands; 2 Centre of Research on Autonomy and Participation, Zuyd University of Applied Sciences, Heerlen, the Netherlands; 3 School for Public Health and Primary Care: CAPHRI; Faculty of Health, Medicine and Life Sciences; Department of Methodology and Statistics, Maastricht University, the Netherlands

## Abstract

**Background:**

Nursing home care for people with dementia is increasingly organized in small-scale and homelike care settings, in which normal daily life is emphasized. Despite this increase, relatively little is known about residents' characteristics and whether these differ from residents in traditional nursing homes. This study explored and compared characteristics of residents with dementia living in small-scale, homelike facilities and regular psychogeriatric wards in nursing homes, focusing on functional status and cognition.

**Methods:**

A cross-sectional study was conducted, including 769 residents with dementia requiring an intensive level of nursing home care: 586 from regular psychogeriatric wards and 183 residents from small-scale living facilities. Functional status and cognition were assessed using two subscales from the Resident Assessment Instrument Minimum Data Set (RAI-MDS): the Activities of Daily Living-Hierarchy scale (ADL-H) and the Cognitive Performance Scale (CPS). In addition, care dependency was measured using Dutch Care Severity Packages (DCSP). Finally, gender, age, living condition prior to admission and length of stay were recorded. Descriptive analyses, including independent samples t- tests and chi-square tests, were used. To analyze data in more detail, multivariate logistic regression analyses were performed.

**Results:**

Residents living in small-scale, homelike facilities had a significantly higher functional status and cognitive performance compared with residents in regular psychogeriatric wards. In addition, they had a shorter length of stay, were less frequently admitted from home and were more often female than residents in regular wards. No differences were found in age and care dependency. While controlling for demographic variables, the association between dementia care setting and functional status and cognition remained.

**Conclusions:**

Although residents require a similar intensive level of nursing home care, their characteristics differ among small-scale living facilities and regular psychogeriatric wards. These differences may limit research into effects and feasibility of various types of dementia care settings. Therefore, these studies should take resident characteristics into account in their design, for example by using a matching procedure.

## Background

The number of people who suffer from dementia is rapidly increasing worldwide, with estimates around 80 million persons in 2040 [1,2]. Its prevalence increases exponentially with age [2]. Dementia is characterized by a variety of symptoms such as cognitive and functional decline and has often a progressive course. The disease burden of dementia is high. It is regarded as the number four cause of disability adjusted life years (DALYs) in older adults (age 60+) [3]. As the disease progresses, nursing home care is often required.

Within nursing home care for people with dementia, there is a trend towards deinstitutionalization [4]. Large nursing homes are transformed into or replaced with small-scale and homelike care settings [5]. In these small-scale care settings, normal daily life is emphasized and residents are encouraged to participate in meaningful activities, centered around the daily household. This opposes against traditional large nursing homes, in which daily life is primarily organized around routines of the nursing home and which have often an institutional character [6].

In various countries, small-scale and homelike care settings have been developed for people with dementia who require a nursing home level of care [5]. Examples include small-scale living in the Netherlands, [6,7] group living in Sweden, [8] group homes in Japan [9] and Green Houses^® ^in the United States [10]. Small-scale living in the Netherlands and group living in Sweden have become widespread models of care. In the Netherlands, it is expected that around 25% of all nursing home care for people with dementia in 2010 will be organized in small-scale living facilities, partly stimulated by the Dutch government. In Sweden, group living facilities housed almost 20% of people with dementia living in institutional care in 2000 [11]. Furthermore, group homes in Japan are increasing rapidly, up to 4775 in 2004 [7].

Despite this transformation, little is known about residents' characteristics in small-scale living facilities and whether these differ from residents in traditional nursing homes. Residents' characteristics are an important factor in exploring whether small-scale living serves a specific subgroup of people with dementia requiring nursing home care. Especially information regarding objective parameters such as functional status and cognition is scarce [5]. Since institutional nursing home care is increasingly organized in small-scale, homelike facilities, knowledge about residents' functional status and cognition is necessary. Some studies investigating effects, including functional status and cognition, had relatively small sample sizes [4,12,13]. Other studies focused on comparison of behavioral problems [14] or only investigated residents in small-scale, homelike facilities without making a comparison with other care facilities [15,16].

In addition, residents' characteristics have important implications for future research, particularly regarding effects and feasibility of dementia care settings. Since randomization in this type of research is difficult to accomplish due to practical and ethical considerations, comparability of resident groups at baseline is essential for interpretation of results. Functional status and cognition appear strongly related to dementia severity [17] and are therefore important baseline residents' characteristics that may influence other outcomes in longitudinal studies, such as quality of life, neuropsychiatric symptoms and social functioning.

This study, therefore, investigated functional status and cognition of residents with dementia requiring a nursing home level of care in two settings: small-scale living and regular psychogeriatric wards in nursing homes. Functional status and cognition were assessed and residents' profiles were constructed. In addition, other resident characteristics such as care dependency, age, gender, length of stay and living condition prior to admission were recorded. These background characteristics were regarded as most important in our study and of potential influence on the outcome measures. The relationship between these variables and the two dementia care facilities was explored in more detail. Findings could contribute to optimal design of and future research into dementia care settings.

## Methods

### Design

A cross-sectional study was conducted in the southern part of the Netherlands, as part of the screening in a longitudinal study investigating effects of small-scale living facilities on residents, family caregivers and nursing staff. The design of this study has been reported elsewhere [6]. The screening was carried out between April 2008 and December 2008. A registered nurse (RN) in charge of the regular psychogeriatric ward or house in a small-scale living facility assessed the residents. Data were collected from questionnaires.

The study was approved by the Medical Ethical Committee of the University Hospital Maastricht and Maastricht University. In addition, local Ethical Committees of participating facilities/wards and their boards gave consent for the study.

### Study population

The study population consisted of 769 residents, all requiring a similar level of intensive nursing home care. Nursing home care in the Netherlands is mainly provided for people with chronic somatic (i.e. physical) diseases, people who require rehabilitation care and people with dementia. They are cared for in specialized somatic, rehabilitation or psychogeriatric wards respectively. This level of care is determined by a standardized assessment procedure, carried out by a government agency. Admission to a nursing home facility, either a small-scale living facility or regular psychogeriatric ward, is based on this assessment and in accordance with the residents' family or legal guardian.

In total, 183 residents in small-scale living facilities were included and 586 residents living in regular psychogeriatric wards of nursing homes. Small-scale living facilities had to fulfill 6 criteria in order to be eligible for the study: 1) a maximum of 8 residents per house or unit, 2) residents, family and staff form a household together, 3) nursing staff perform multiple tasks, such as medical and personal care, organizing activities and domestic chores 4) a small, fixed team of nursing staff who care for the residents 5) daily life is largely organized by residents, their family members and nursing staff and 6) the facility resembles a typical homelike environment [6]. Five small-scale living facilities were selected and included in the study, with 28 houses in total.

Regular psychogeriatric wards in nursing homes were selected based on the following criteria: 1) a minimum of 20 residents per ward, 2) staff have differentiated tasks, focusing on residents' medical and personal care and 3) the routines of the nursing home largely determine residents' daily life. In total, 7 nursing homes were selected and participated in the study, with 21 psychogeriatric wards.

### Measures

#### Functional status

Functional status was measured using the Activities of Daily Living-Hierarchy (ADL-H) subscale [18,19] from the Resident Assessment Instrument Minimum Data Set (RAI-MDS), version 2.1 [20]. This 7 category hierarchical scale comprises 4 items assessing ADL activities personal hygiene, toilet use, locomotion and eating. These items are found most consistent with various stages of loss of functioning: early (personal hygiene), middle (toilet use and locomotion) and late (eating) loss of functioning[18]. Scores range from 0 (independent) to 6 (totally dependant).

#### Cognition

Cognition was assessed using the Cognitive Performance Scale (CPS) [19,21], another subscale from the RAI-MDS, version 2.1 [20]. The CPS includes 5 items, addressing cognitive and communication aspects (short-term memory, decision making and making oneself understood), presence of coma and eating dependency. The items form a hierarchical scale, consisting of 7 categories and ranging from 0 (intact) to 6 (very severe impairment). Based on a decision tree, total CPS scores are calculated [21]. Previous research has shown that CPS scores correspond strongly to scores on the widely used Mini-Mental State Examination [19,21-24].

#### Care dependency

Care dependency was assessed using the Dutch Care Severity Packages (DCSP) scores (in Dutch 'ZorgZwaartePakketten' (ZZPs)). This is a standardized assessment which is used in all Dutch nursing homes to assess the amount and type of care that a resident needs. It consists of a 54-item questionnaire, covering several care domains, such as (psycho)social functioning, personal and nursing care, mobility and behavioral problems. An algorithm is used to calculate DCSP scores. There are 10 DCSP scores available in nursing home care, which are divided in three categories: long-term care (DCSP scores 1 to 8), care aimed at rehabilitation (DCSP score 9) and end-of-life care (DCSP score 10) [25]. Within long-term care, a higher DCSP score indicates a higher care dependency.

#### Background characteristics

Residents' age, gender, living condition prior to admission (e.g. home, residential care or nursing home) and length of stay were recorded using a questionnaire. Furthermore, it was assessed whether residents had a (probable) diagnosis of dementia (yes or no).

Functional status and cognition were assessed on-site by a registered nurse (RN) in charge of the regular psychogeriatric ward or house in a small-scale living facility, specifically for this study. Care dependency and background characteristics were derived from residents' record by the RN. All care dependency scores were recently assessed prior to data collection as part of an annual registration.

#### Statistical Analysis

The Statistical Package for Social Sciences (SPSS) version 15.0 was used for data analysis. Descriptive statistics were computed to present residents' characteristics per setting. In addition to mean scores on functional status and cognition, a residents' profile was constructed for detailed analyses. To obtain a profile for residents regarding functional status and cognition, scores on these measures were dichotomized. For cognition, the three highest scores (i.e. 4-6) were regarded as a low cognitive level; the remaining categories (i.e. 0-3) formed a relatively high level of cognition. For functional status, the three lowest scores (i.e. 0-2) were combined as a relatively high functional status; the 4 other categories (i.e. 3-6) were considered as a low level of functional status[26]. Cross-tabs were calculated to compare profiles between residents in small-scale living and regular psychogeriatric wards.

Differences between the two dementia care facilities were tested using independent samples t-tests for the variables functional status, cognition, age and length of stay; care dependency, gender and living condition prior to admission were analyzed using chi-square-tests. Since length of stay was not normally distributed in both groups, a log transformation was used in the analyses.

To explore the relationship between residents' characteristics and care setting in more detail, multivariate logistic regression analysis was performed, with type of care setting (small-scale living versus regular psychogeriatric ward) as dependent variable and residents' characteristics as independent variables. In all tests, a significance level α of .05 was used.

## Results

Table 1 presents descriptive and test statistics for all residents' measurements in both dementia care settings.

**Table 1:** Residents' characteristics: small-scale living and regular psychogeriatric wards

	Small-scale Living	Regular Wards	*p *- Value
Age, mean ± SD (range)	82.72 ± .57 (61-101)	82.50 ± .30 (57-101)	.73*

Gender, n (%)			.01^†^

Male	36 (19.7)	175 (29.9)	

Female	146 (79.8)	407 (69.5)	

Unknown	1 (.5)	4 (.4)	

Living condition prior to admission			.00^†^

Home	53 (29.0)	362 (61.8)	

Home for the elderly	31 (16.9)	61 (10.4)	

Nursing home	77 (42.1)	87 (14.8)	

Other/Unknown	22 (12.0)	76 (13.0)	

Length of Stay^‡^, mean ± SD (range)	15.43 ± .57 (1-37)	32.56 ± 1.09 (1-190)	.00*

Diagnosis of dementia			.33^†^

Yes	176 (96.2)	556 (94.9)	

No	2 (1.1)	10 (1.7)	

Unknown	5 (2.7)	20 (3.4)	

Care dependency^§^			.33^†,¶^

DCSP ≥ 1 ≤ 5	121 (66.2)	386 (65.9)	

DCSP ≥ 6 ≤ 8	46 (25.1)	177 (30.2)	

DCSP = 9	3 (1.6)	1 (.2)	

DCSP = 10	0 (.0)	12 (2.1)	

Unknown	13 (7.1)	10 (1.6)	

Cognition^#^, mean ± SD (range)	3.52 ± .11 (0-6)	4.40 ± .06 (0-6)	.00*

Functional Status^#^, mean ± SD (range)	3.26 ± .13 (0-6)	4.14 ± .06 (0-6)	.00*

* Data were analyzed using independent t-tests.

^†^Data were analyzed using Chi-Square tests.

^‡ ^as measured in months

^§ ^DCSP = Dutch Care Severity Package; scores 1-5 represent a relatively low level of care dependency, scores 6-8 represent a relatively high level of care dependency, score 9 represents rehabilitation care and score 10 represents terminal care.

^¶^Chi-square is calculated for two groups: DCSP ≥ 1 ≤ 5 and DCSP ≥ 6 ≤ 8 since categories 9 and 10 contained too little cases for valid testing.

# Normal range: 0 - 6; a lower score indicates a better performance.

### Background characteristics

Significant differences (all p < .01) were found in gender, living condition prior to admission and length of stay. Relatively more women lived in small-scale living facilities compared with traditional nursing homes. Furthermore, more residents in regular psychogeriatric wards had lived at home prior to admission, whereas residents in small-scale living facilities had more often been transferred from a regular ward. In addition, residents in traditional nursing homes had lived longer at their ward than those in small-scale living facilities (see Table 1). No differences were found for age, diagnosis of dementia and care dependency.

### Functional status and cognition

Significant differences (all p < .01) were found in both functional status and cognition. Residents in small-scale living facilities had a better cognitive and functional status, as reflected in lower CPS and ADL-H scores than residents of traditional nursing homes. Table 2 presents residents' profile regarding cognition and functional status. It shows that residents with both a high level of cognition and functional status were overrepresented in small-scale living facilities compared with regular psychogeriatric wards (30.7% and 10.6% respectively). Additionally, residents with a relatively low cognitive and functional status were overrepresented in regular psychogeriatric wards: 66.0% compared with 42.5% in small-scale living.

**Table 2:** Cognition and ADL profile: small-scale living and regular psychogeriatric wards compared

	Functional Status, n (%)		
	
	High (ADL-H score 0-2)	Low (ADL-H score 3-6)	Total
Small-scale living			

Cognition, n (%)			

High (CPS score 0-3)	55 (30.7)	42 (23.5)	97 (54.2)

Low (CPS score 4-6)	6 (3.4)	76 (42.5)	82 (45.8)

Total	61 (34.1)	118 (65.9)	179 (100)

Regular psychogeriatric wards			

Cognition, n (%)			

High (CPS score 0-3)	62 (10.6)	126 (21.6)	188 (32.2)

Low (CPS score 4-6)	10 (1.7)	385 (66.0)	395 (67.8)

Total	72 (12.3)	511 (87.7)	583 (100)

In both types of facilities, the majority of residents had a low functional status, although for regular psychogeriatric wards this is far more prominent with a total of 87.7% having a low functional status versus 65.9% in small-scale living. Level of cognition was almost equally distributed in small-scale living, with slightly more residents having a relatively high cognition (i.e. 54.2%). However, in regular psychogeriatric wards, residents with a relatively high cognitive level were outnumbered: approximately 2 out of 3 residents (67.8%) had a low cognitive level.

### Multivariate logistic regression

Table 3 shows the results of the final regression model. Nagelkerke R^2 ^was .31. R^2 ^is a measure that indicates how well the dependent variable, in this case dementia care setting, can be determined by the independent variables and ranges from zero to one [27].

**Table 3:** Results of logistic regression analysis, final model*

Residents' characteristics	B (SE)	Odds Ratio	95% CI for Odds Ratio
Gender^†^	.89 (.25)	2.42	1.49 - 3.94

Living condition prior to admission^‡^			

Home for the elderly	-1.39 (.29)	.25	.14 - 0.45

Nursing home	-2.06 (.24)	.13	.08 - .21

Other	.85 (.51)	2.33	.85 - 6.36

Length of stay	.63 (.23)	1.88	1.20 - 2.96

Cognition	.26 (.09)	1.30	1.09 - 1.54

Functional status	.24 (.09)	1.27	1.07 - 1.50

* Nagelkerke R^2 ^= .31; dependent variable is dementia care setting: small-scale living facility = 0, regular psychogeriatric ward = 1.

^† ^Gender: Female = 0, Male = 1.

^‡ ^Reference group is 'Home'.

Regression analysis confirmed significant associations (all p < .01) for dementia care setting and functional status, cognition, gender, living condition prior to admission and length of stay. The chance of living in a regular psychogeriatric ward increased with almost 30% per one point increase on the scales measuring cognition and ADL. This means that residents who were more cognitive and ADL impaired, lived more often in a regular psychogeriatric ward. In addition, the chance that men lived at a regular psychogeriatric ward was almost 2.5 times higher than for women (range 1.5 - 3.9). Residents admitted from a home for the elderly or another regular psychogeriatric ward had a higher chance of being admitted at a small-scale living facility, compared with residents admitted directly from home. Finally, the chance of living on a regular psychogeriatric ward increased with around 88% per 10 months of length of stay.

## Discussion and Conclusions

This study showed that residents' characteristics differ in small-scale living facilities and regular psychogeriatric wards, although all residents required a similar nursing home level of care. Residents in small-scale living facilities had a higher cognitive and functional status than residents in regular wards. Demographic characteristics such as living condition prior to admission and length of stay could explain these results to some extent. Length of stay in small-scale living facilities was inevitably shorter, since these are relatively new facilities (newest facility was open for one year), whereas regular nursing home wards are located in long established facilities. This explains the large difference (i.e. 17 months) in mean length of stay between the two care settings. However, while controlling for this and other demographic variables, the association remained between dementia care setting and cognition and functional status. Although some studies have found similar results regarding functional status [12,14,28] and cognition [13], other studies did not find significant differences [4,29].

An explanation for our findings may be that selection has occurred in allocating residents to small-scale living facilities, despite similar admission criteria for both dementia care settings as determined by a standardized assessment procedure performed by a governmental agency. Most of these residents were transferred from a regular psychogeriatric ward. As residents in small-scale living had better cognitive and ADL performance, it seems that residents with the best cognitive and functional abilities were selected for the small-scale living facilities. A recent study by te Boekhorst et al. (2009) confirms this explanation [7]. They found that residents admitted in small-scale living were in a slightly earlier stage of dementia than residents admitted in traditional nursing homes, as reflected in significantly higher cognitive performance and functional abilities.

A selection process is probably related to the innovative concept of small-scale living facilities. Although small-scale living is currently expanding in the Netherlands, these facilities are still relatively new compared with traditional nursing homes. Over time, residents' characteristics may change resulting in an increased care dependency and decreased cognitive and functional status. Research conducted in Sweden supports this assumption. In Sweden, group living is a long-established dementia care setting, in which residents have become more ADL dependent over the years [11,16]. These results support a clinical experience in Sweden that over time, residents were admitted in a later stage in their dementia [16]. However, our study identified that already 42.5% of residents in small-scale living had a low level of cognition and functional status. These results highlight the importance of research into suitability of small-scale living for residents with more cognitive and functional impairments.

In our study, the level of care dependency, as measured with DCSP scores, did not differ between the two settings. This is in line with the standardized assessment procedure to determine the level of care: all residents in our study require a similar intensive nursing home level of care. However, we found that residents in small-scale living facilities were more independent in ADL and had a better cognitive performance. Since we derived DCSP scores from the medical record, this might not correspond completely in time with the assessment of ADL and cognition during the screening. Therefore residents might have deteriorated due to the progressive nature of their disease which could explain the differences. Moreover, care dependency constitutes more than just cognition and ADL dependency, including behavioral problems for example. In the DCSP scores, behavioral problems are incorporated among others, where a higher score indicates more (behavioral) problems. However, previous research suggested that DCSP items relating to behavior were possibly more difficult to interpret and had a lower reliability than other DCSP items [30]. The overall DCSP scores' validity or reliability was not studied. More research is needed to confirm that DCSP scores are a valid and reliable measure of care dependency and how this measure is related to other validated measures of care dependency.

Additionally, health care policy and economic issues might have had an influence, since financing of care settings is based on these DCSP scores. An adequate score on the DSCP measure might have been a selection criterion for intake in a small-scale living facility, without residents really being as care-dependant as in a regular nursing home ward. Most residents in our sample, approximately two third in both care settings, had a relatively low level of care dependency (DCSP scores 1-5). It might be that for small-scale living facilities, this is an underestimation and that actually residents now classified as having a relatively high care dependency (DSCP scores 6-8) are actually in a lower need of care.

Some limitations regarding this study must be considered. This study focused on cognition and functional status and therefore assessed only a limited number of variables. Other relevant characteristics such as behavioral problems and social functioning need to be investigated as well. Additionally, residents in other care settings could be included, for example residential care, to cover the whole continuum of dementia care in the Netherlands. Furthermore, a cross-sectional design was used, since this study's objective was to compare residents in two dementia care settings. This design limits causal interpretation of our results. For example, it might be possible that at admission ADL and cognition were the same for residents in both care settings, which may imply a positive effect of small-scale living facilities. However, in our sample no standardized information regarding these patient characteristics at admission was present, which is a drawback. Therefore no inferences can be drawn regarding effects of small-scale living facilities regarding the variables ADL and cognition. Longitudinal research is needed to investigate effects of dementia care setting on residents, addressing several important outcome measures such as quality of life, functional status, behavioral problems and social functioning. This is important, since dementia care settings are increasingly directed towards small-scale and homelike facilities. A few studies have been reported regarding these measures showing promising results [4,7,12,13,28]. However, methodological limitations such as small sample sizes, differences at baseline between groups or a relatively short follow-up period, hinder interpretation of results.

Our results suggest that functional status and cognition of residents living in small-scale, homelike facilities is better than in regular psychogeriatric wards of nursing homes. These differences in baseline characteristics have implications for research and practice. Effectiveness of new dementia care settings is hard to predict. Research focusing on effects of care settings on residents, family members and nursing staff should take baseline differences in residents' characteristics into account, since these could influence outcome measures. Matching of residents based on a profile of functional status and cognition could form a solution for this challenge. This procedure will increase a study's internal validity and therefore enhance the prognostic comparability of the study groups. In addition, statistical analyses can be used to correct for remaining baseline differences between groups.

Furthermore, development of small-scale living facilities may influence daily practice in more traditional nursing homes. Our results suggest that residents with better cognitive and functional abilities were transferred from traditional nursing homes. As a result, care dependency in traditional nursing homes may increase. Our results highlight the importance of research into optimal environments in the continuum of dementia care.

## Competing interests

The authors declare that they have no competing interests.

## Authors' contributions

All authors were involved in the analysis and interpretation of data, critically reviewed the manuscript, read and approved the final manuscript. HV, EvR, SMGZ, GIJMK and JPHH are involved in the concept and study design, acquisition of subjects and data. TA gave advices on the statistical analysis. All authors read and approved the final manuscript.

## Pre-publication history

The pre-publication history for this paper can be accessed here:

http://www.biomedcentral.com/1472-6963/10/30/prepub
